# Lesion characteristics and procedural complications of chronic total occlusion percutaneous coronary intervention in patients with prior bypass surgery: A meta‐analysis

**DOI:** 10.1002/clc.23766

**Published:** 2022-01-06

**Authors:** Yuchen Shi, Songyuan He, Jesse Luo, Wen Jian, Xueqian Shen, Jinghua Liu

**Affiliations:** ^1^ Center for Coronary Artery Disease (CCAD), Beijing Anzhen Hospital, and Beijing Institute of Heart, Lung and Blood Vessel Diseases Capital Medical University Beijing China

**Keywords:** chronic total occlusion, coronary artery bypass graft, meta‐analysis, percutaneous coronary intervention

## Abstract

Coronary artery bypass graft (CABG) accelerates the prevalence of native coronary chronic total occlusion (CTO), and this kind of CTO shows extensive challenging and complex atherosclerotic pathology. As a result, the procedural success rate of percutaneous coronary intervention (PCI) is inferior to another kind of lesions. The present meta‐analysis aims to compare the lesion characteristics and procedural complications of CTO‐PCI in patients with or without prior CABG. A total of 8 studies, comprising of 13439 patients, published from inception to August 2021 were included in this meta‐analysis. Results were pooled using random effects model and are presented as odds ratio (OR) with 95% confidence intervals (95% CIs). From the 13439 patients enrolled, 3349 (24.9%) patients had previous CABG and 10090 (75.1%) formed the control group in our analysis. For the clinical characteristic, compared to the non‐CABG patients, prior CABG patients were older (OR, 3.98; 95% CI, 3.19–4.78; *p* < .001; *I* ^2^ = 72%), had more male (OR, 1.30; 95% CI, 1.14–1.49; *p* < .001; *I* ^2^ = 6%), diabetes mellitus (OR, 1.54; 95% CI, 1.36–1.73; *p* < .001; *I* ^2^ = 37%), dyslipidemia (OR, 1.89; 95% CI, 1.33–2.69; *p* < .001; *I* ^2^ = 81%), hypertension (OR, 1.88; 95% CI, 1.46–2.41; *p* < .001; *I* ^2^ = 71%), previous myocardial infarction (OR, 1.94; 95% CI, 1.48–2.56; *p* < .001; *I* ^2^ = 85%), and previous PCI (OR, 1.74; 95% CI, 1.52–1.98; *p* < .001; *I* ^2^ = 22%). Non‐CABG patents had more current smoker (OR, .45; 95% CI, 0.27–0.74; *p* < .001; *I* ^2^ = 91%). BMI (OR, −0.01; 95% CI, −0.07–0.06; *p* = .85; *I* ^2^ = 36%) were similar in both groups. For lesions location, the right coronary artery (RCA) was predominant target vessel in both groups (50.5% vs 48.7%; *p*＝.49), although, the left circumflex (LCX) was more frequently CTO in the prior CABG group (27.3% vs 18.9%; *p*＜.01), while left anterior descending artery (LAD) in non‐CABG ones (16.0% vs 29.1%; *p*＜0.01). For lesions characteristics, prior CABG patients had more blunt stump (OR, 1.71; 95% CI, 1.46–2.00; *p* < .001; *I* ^2^ = 40%), proximal cap ambiguity (OR, 1.45; 95% CI, 1.28–1.64; *p* < .001; *I* ^2^ = 0.0%), severe calcifications (OR, 2.91; 95% CI, 2.19–3.86; *p* < .001; *I* ^2^ = 83%), more bending (OR, 3.07; 95% CI, 2.61–3.62; *p* < .001; *I* ^2^ = 0%), lesion length > 20 mm (OR, 1.59; 95% CI, 1.10–2.29; *p* = .01; *I* ^2^ = 83%), inadequate distal landing zone (OR, 1.95; 95% CI, 1.75–2.18; *p*＜.001; *I* ^2^ = 0.0%), distal cap at bifurcation (OR, 1.65; 95% CI, 1.46–1.88; *p* < .001; *I* ^2^ = 0.0%), and higher J‐CTO score (SMD, 0.52; 95% CI, 0.42–0.63; *p* < .001; *I* ^2^ = 65%). But side branch at proximal entry (OR, 0.88; 95% CI, 0.72–1.07; *p* = .21; *I* ^2^  = 45%), in‐stent CTO (OR, 0.99; 95% CI, 0.86–1.14; *p* = .88; *I* ^2^ = 0.0%), lack of interventional collaterals (OR, 0.80; 95% CI, 0.55–1.15; *p* = .23; *I* ^2^ = 78%), and previously failed attempt (OR, 0.73; 95% CI, 0.48–1.11; *p* = .14; *I* ^2^ = 89%) were similar in both groups. For complication, prior CABG patients had more perforation with need for intervention (OR, 1.91; 95% CI, 1.36–2.69; *p* < 0.001; *I* ^2^ = 34%), contrast‐induced nephropathy (OR, 3.40; 95% CI, 1.31–8.78; p = .01; *I* ^2^ = 0.0%). Non‐CABG patents had more tamponade (OR, 0.25; 95% CI, 0.09–0.72; *p* = .01; *I* ^2^ = 0.0%), and the major bleeding complication (OR, 1.18; 95% CI, 0.57–2.44; *p* = .65; *I* ^2^ = 0%) were no significant difference in both groups. In conclusion, Patients with prior CABG undergoing CTO‐PCI have more complex lesion characteristics, though procedural complication rates were comparable.

## 1 INTRODUCTION

Coronary chronic total occlusion (CTO) is characterized by complete occlusion of anterograde blood flow (TIMI grade 0 flow) in the coronary artery, presented more than 3 months. CTO percutaneous coronary intervention (PCI) represents one of the most advanced fields of interventional cardiology. Owing to the complexity of CTO‐PCI, only 10%–15% of CTO patients attempted to receive interventional revascularization, and more patients are undergoing coronary artery bypass grafting (CABG) surgery.^1^ CABG significantly improved the long‐term clinical outcomes of complex coronary lesions indeed. However, CABG itself can accelerate the development of native coronary artery atherosclerosis, leading to development of a new CTO in up to 43% of the bypassed native arteries as early as 1 year after CABG.^2^ And the prevalence of native artery CTO in patients undergoing coronary angiography reaches 54% in the post‐CABG population, higher than in patients without CABG.^3^ Moreover, repeat CABG was related to worse long‐term clinical outcomes as compared to initial CABG. And saphenous vein graft PCI also has high rates of failure.^4^ Therefore, for these cases, the native arteries CTO‐PCI was preferred as a revascularization strategy.

Although several experts have regarded previous CABG as a predictor of technical failure in CTO‐PCI.^5^ Patients who underwent previous CABG have exhibited more inflammation, fibrosis, calcification, and negative remodeling in CTO lesions compared with CABG‐naïve patients for pathologic examination.^6^ Consequently, previous CABG has been included as a risk factor for procedural technical failure in the Registry of CrossBoss and Hybrid procedures in France, the Netherlands, Belgium, and the United Kingdom (RECHARGE) score.^5^ Over the past decade, thanks to the remarkable update of revascularization techniques, equipment, and contemporary algorithms in CTO‐PCI procedures, CTO‐PCI success rates have approached 90% for experienced operators currently.^7^ Thus, PCI has emerged as a promising alternative treatment for CTO revascularization in post‐CABG patients.

However, the lesion characteristics of CTO‐PCI for a native coronary artery in previous CABG status have been sparsely studied, and it remains unclear whether the higher procedural difficulty encountered during CTO‐PCI in patients who have undergone CABG is also mirrored by worse complications. Some recent studies investigated the lesion characteristics of CTO‐PCI in previous CABG patients; however, the results were inconsistent. Furthermore, whether the higher procedural complexity encountered during CTO‐PCI in previous CABG patients translated into major complications is yet to be clarified. Given the increasing prevalence of patients with CTO after CABG, along with the development of novel interventional approaches, characterization, and complications of these patients gained further importance. To shed further light on this issue, we conducted a systematic review and meta‐analysis to assess clinical and procedural characteristics as well as in‐hospital major complications of CTO‐PCI in patients with and without CABG.

## 2 METHODS

### 2.1 Data sources and search strategy

Two investigators (Yuchen Shi and Songyuan He) independently performed a comprehensive search of the PubMed, Embase, and Cochrane Library databases from inception to August 15, 2021, using the following search terms: (1) chronic total occlusion, CTO, and coronary occlusion; (2) percutaneous coronary intervention and PCI; (3) coronary artery bypass, coronary bypass, bypass surgery, and CABG.

### 2.2 Study selection

Studies were included when the following were satisfied: (1) studies with patients treated by CTO‐PCI. CTO was defined as a TIMI grade 0 flow in coronary artery for more than 3 months; (2) studies with comparisons of CTO‐PCI in patients with and without prior CABG; (3) studies that reported the angiographic characteristics and in‐hospital complications in both groups. And case reports, reviews, notes, letters, commentaries, and editorials were excluded.

### 2.3 Endpoints

The angiographic characteristics of the enrolled studies included blunt stump, degree of calcification, bending, lesion length, previously failed attempt, proximal cap ambiguity, situation of interventional collaterals, side branch at proximal entry, in‐stent CTO, inadequate distal landing zone, distal cap at bifurcation, and J‐CTO score. The in‐hospital clinical complication included perforation, tamponade, major bleeding, and contrast‐induced nephropathy.

### 2.4 Statistical analysis

For dichotomous data, the random‐effects model with the Mantel‐Haenszel method was used as a summary statistic to calculate the pooled odds ratio (OR) with the 95% confidence intervals (95% CIs). For continuous data, standard mean differences (SMD) calculated according to the inverse‐variance method was used. Statistical heterogeneity was assessed using Cochrane's Q via the chi‐square test and further quantified with the *I* ^2^ statistic. And 25%, 50%, and 75% indicated low, moderate, and high heterogeneity, respectively. All statistical analyses were conducted using Review Manager 5.4.1 version (RevMan, The Cochrane Collaboration). As less than 10 studies were included in our meta‐analysis, funnel plots were not used to assess publication bias.

## 3 RESULTS

### 3.1 Characteristics of the studies and patients included

**Figure 1:**
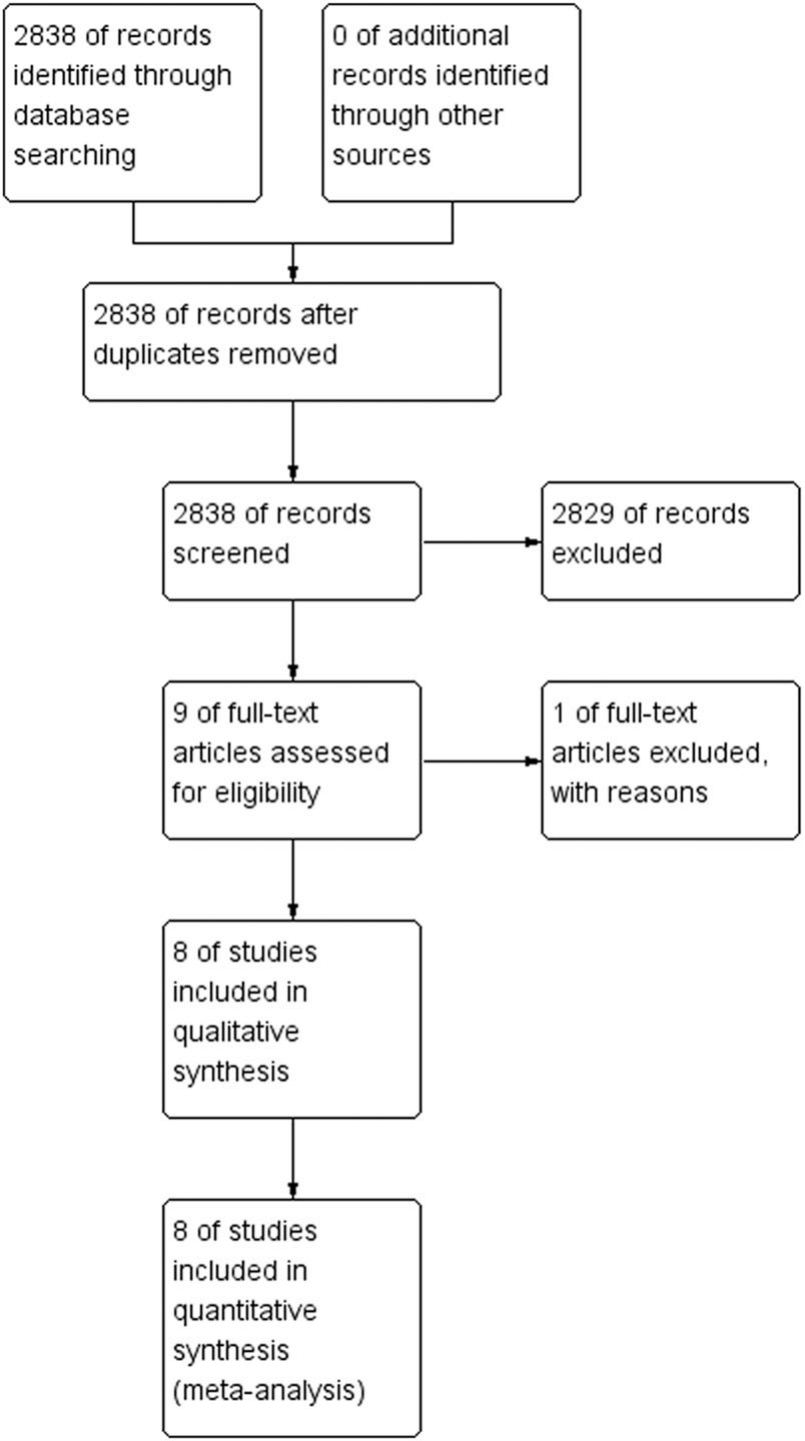
Flow diagram of study selection

Figure 1 shows the flowchart of the study selection. As a result, a total of 2838 studies were identified through electronic searches. Then, 2829 studies were excluded after reading the titles and abstracts. The remaining nine studies were evaluated by reading the full texts. Eventually, eight studies comprising a total of 13 439 patients met the inclusion criteria and were included in qualitative synthesis and meta‐analysis.^8^, ^9^, ^10^, ^11^, ^12^, ^13^, ^14^, ^15^ The baseline clinical characteristics of the patients included in this meta‐analysis are summarized in Table 1A. Out of the 13 439 patients enrolled, 3349 (24.9%) presented with previous CABG and 10 090 (75.1%) formed the control group for our analysis. Patients who had undergone CABG were older (68.0 ± 8.8 years vs. 64.3 ± 10.3 years; *p* < .00001), were mostly men (87.2% vs. 84.0%; *p* < .0001), but the BMI had no different (29.6 ± 5.4 vs. 29.2 ± 6.0; *p* = .85). For past medical history, there were more previous myocardial infarctions (54.7% vs. 36.7%; *p* < .00001) and previous PCI (65.7% vs. 48.1%; *p* < .00001). For risk factors, post‐CABG patients had a higher prevalence of diabetes mellitus (45.2% vs. 33.8%; *p* < .00001), hypertension (88.3% vs. 76.7%; *p* < .00001) and dyslipidemia (89.4% vs. 76.5%; *p* = .0004), but the current smoking prevalence was lower (13.6% vs. 24.5%; *p* = .002).

**Table 1A:** The characteristics of patients included in this meta‐analysis

Study	Azzalini, 2018		Budassi, 2020		Dautov, 2016		Michael, 2013		Nikolakopoulos, 2020		Tajti, 2019		Teramoto, 2014		Toma, 2016		*P* value
Study type	Retrospective cohort study		Retrospective cohort study		Retrospective cohort study		Retrospective cohort study		Retrospective cohort study		Retrospective cohort study		Retrospective cohort study		Retrospective cohort study		
Characteristics	Prior CABG	No prior CABG	Prior CABG	No prior CABG	Prior CABG	No prior CABG	Prior CABG	No prior CABG	Prior CABG	No prior CABG	Prior CABG	No prior CABG	Prior CABG	No prior CABG	Prior CABG	No prior CABG	
Study total size (*n*)	401	1657	217	1035	175	295	508	855	502	1082	1101	2317	153	1139	292	1710	
Age (y)	69.2 ± 8.0	64.3 ± 10.6	68.5 ± 8.5	64.9 ± 10.7	70 ± 7	64 ± 11	67.7 ± 9.0	63.3 ± 10.4			67.3 ± 9.3	63.2 ± 10.2	68.2 (62.4–74.6)	66.0 (58.2–73.6)	68 ± 9	65 ± 11	1E−05
Male sex (%)	92	87	86.2	85.5	86	77	86.2	84.4			87.1	83.8	82	82	88	83	0.0001
Body mass index (kg/m^2^)	28.8 ± 5.1	28.6 ± 7.3	28.3 ± 3.9	28.5 ± 4.8	29 ± 5	30 ± 6					30.6 ± 5.8	30.7 ± 6.3			28.5 ± 4.4	28.1 ± 4.4	0.85
Diabetes (%)	48	35	31.3	25.5	52	30	44.3	36.8			48.8	38.6	42	37	39	28	1E−05
Dyslipidemia (%)	91	78	78.3	64.7			96	92.6			95.3	87.7	35	37	91	85	0.0004
Hypertension (%)	87	74	72.4	59.3	93	75	92.6	87.2			93.7	88	59	61	90	81	1E−05
Current smoker (%)	12	31	7.4	24.6	7	23					20.5	29.8	18	25	7	22	0.002
Previous myocardial infarction (%)	56	43	51.2	36.6	65	51	44.9	39.8			56.4	42.8			48	21	1E−05
Previous PCI (%)	73	58	62.5	55.9	76	67	43.4	40.8			73.6	60.1			23	14	1E−05

### 3.2 Angiographic characteristics

#### 3.2.1 Lesions location

All studies reported the lesions' location, which involved 3422 lesions in the post‐CABG group and 10 430 lesions in the no‐CABG group. The CTO lesions ratio distributions are shown in Table 1B. The right coronary artery (RCA) was the main target vessel in both groups (50.5% vs. 48.7%; *p* = .49), although, the left circumflex (LCX) was more frequently CTO in the prior CABG group (27.3% vs. 18.9%; *p* < .00001), while left anterior descending artery (LAD) in non‐CABG patients (16.0% vs. 29.1%; *p* < .00001).

**Table 1B:** The lesions location of patients included in this meta‐analysis

Study	Azzalini, 2018		Budassi, 2020		Dautov, 2016		Michael, 2013		Nikolakopoulos, 2020		Tajti, 2019		Teramoto, 2014		Toma, 2016		*P* value
CTO target vessel	Prior CABG	No prior CABG	Prior CABG	No prior CABG	Prior CABG	No prior CABG	Prior CABG	No prior CABG	Prior CABG	No prior CABG	Prior CABG	No prior CABG	Prior CABG	No prior CABG	Prior CABG	No prior CABG	
Right coronary artery (%)	53	49	67.3	59.1	57	48	56.2	54.7	55.94	53.95	56.2	55.1	45	43	44	47	0.49
Circumflex (%)	26	20	22.6	14.6	20	29	27.4	20.1	25.2	16.2	26.1	16.7	31	22	37	23	1E−05
Left anterior descending artery (%)	21	31	8.3	26.3	16	10	14.2	25	16.8	29.47	16.6	27.8	22	34	15	30	1E−05
Other (%)	0	0	1.8	0	7	13	2.2	0.2	0.41	0.28	1.1	0.4	2	0.3	5	0.1	0.0001

#### 3.2.2 Blunt stump

Four studies involving a total of 1914 patients in the prior CABG group and 5352 patients in non‐CABG patients, reported the incidence of a blunt stump in those allocated to pCABG 56.1% (1074 of 1914) or nCABG 44.2% (2367 of 5352) treatment. The pooled outcomes revealed that patients with previous CABG undergoing CTO‐PCI often have more blunt stump compared with the no‐CABG group (OR: 1.71, 95% CI: 1.46–2.00, *p* < .00001). And low heterogeneity was found among these studies (*I* ^2^ = 40%, *p* = .17; Figure 2A).

Figure 2Forest plot for: (A) blunt stump; (B) side branch at proximal entry; (C) proximal cap ambiguity; (D) calcifications; (E) bending; (F) lesion length; (G) in‐stent CTO; (H) lack of interventional collaterals; (I) inadequate distal landing zone; (J) distal cap at bifurcation; (K) previously failed attempt; (L) J‐CTO score

**Figure:**
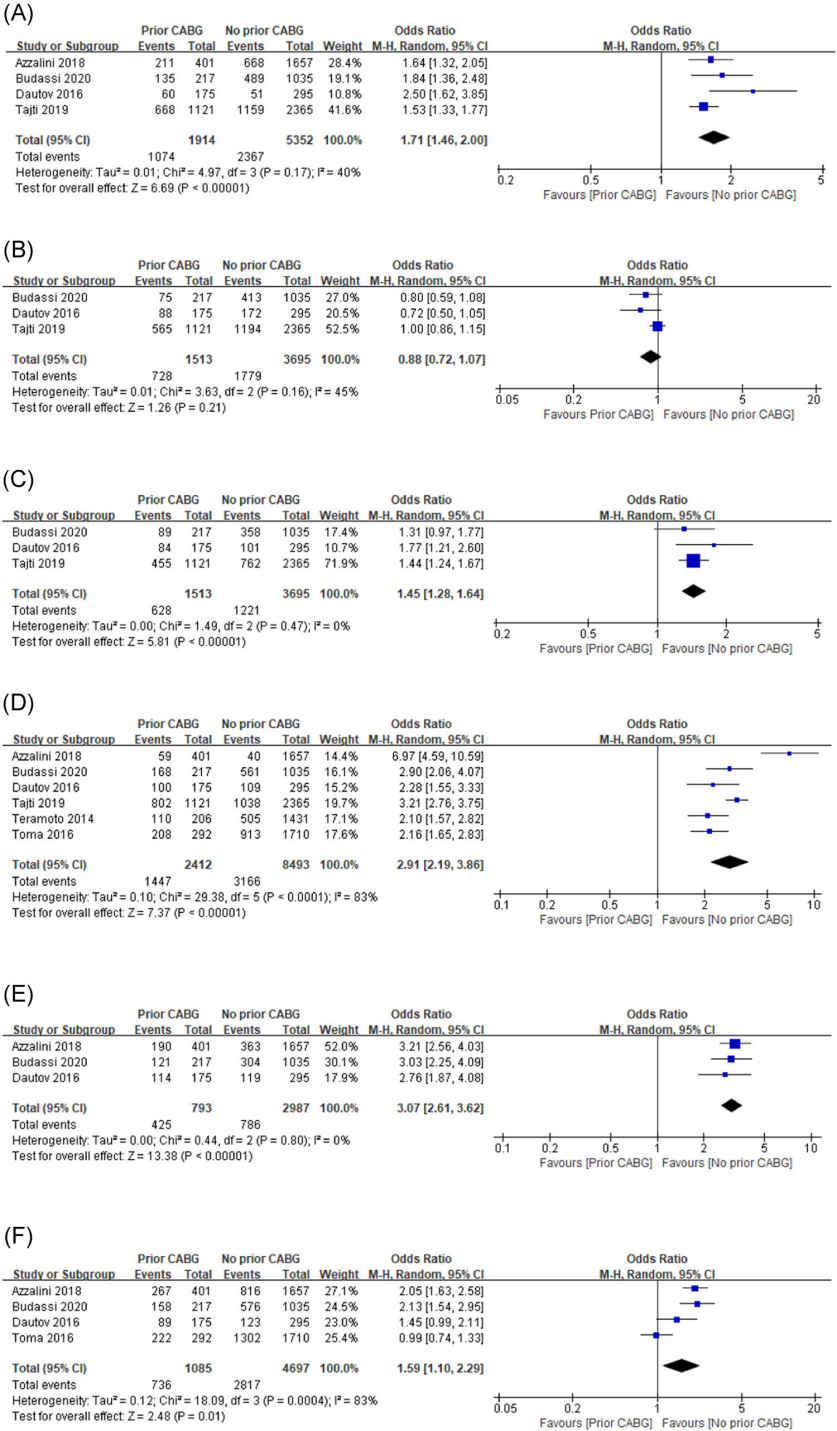

**Figure:**
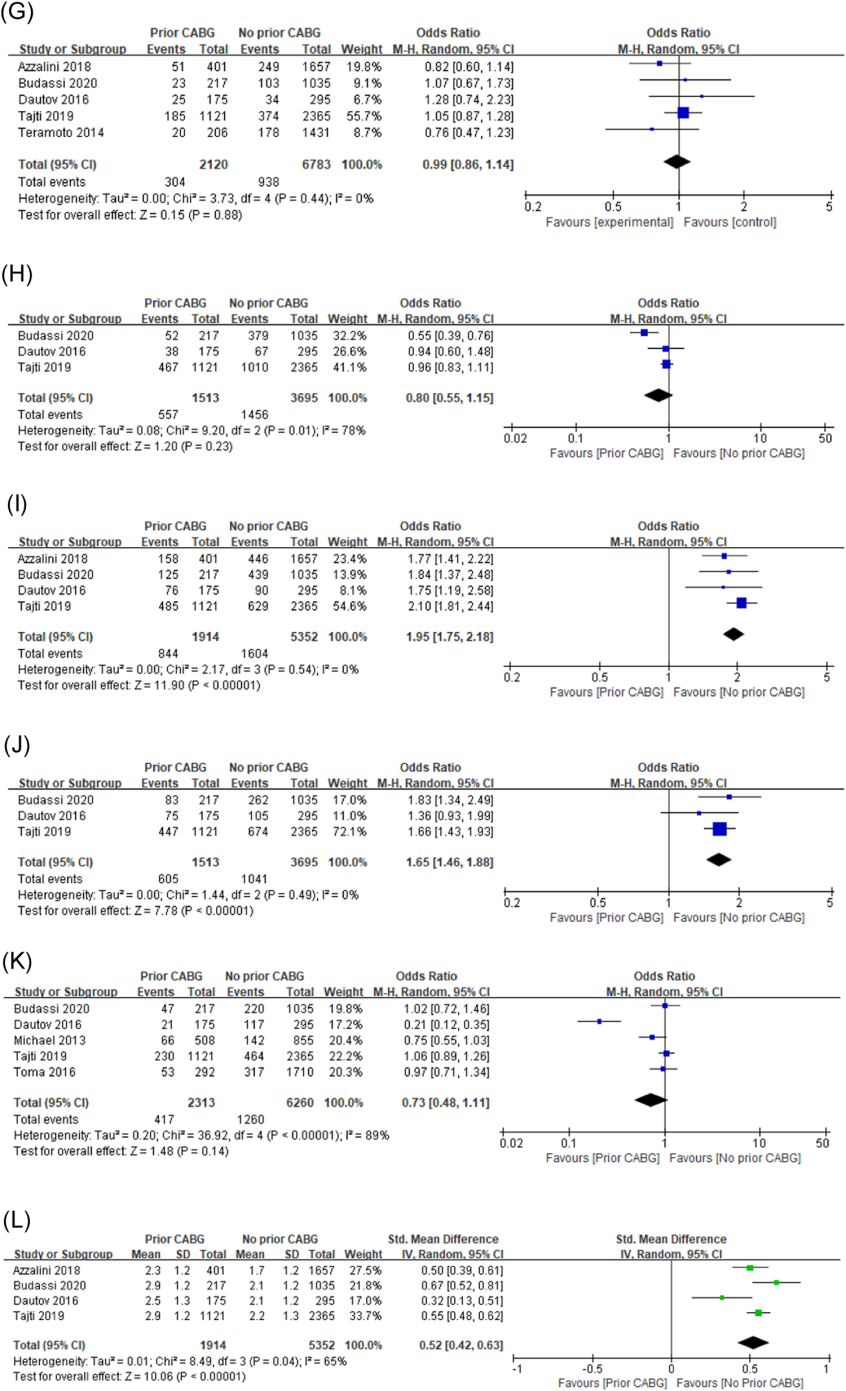

#### 3.2.3 Side branch at proximal entry

Figure 2B pooled analysis of three studies from 5208 patients revealed 2507 events (48.1%): per treatment, the event rate was 48.1% (728 of 1513) in the prior CABG group and 48.1% (1779 of 3695) in the non‐CABG group. No significant difference was observed between the two groups and the overall OR was 0.88 (95% CI: 0.72–1.07, *p* = .21), with moderate heterogeneity (*I* ^2^ = 45%, *p* = .16).

#### 3.2.4 Proximal cap ambiguity

Proximal cap ambiguity in angiographic makes PCI more complex. Only three studies involved 5208 participants and 1849 events (35.5%), but a positive result was found. Regarding treatment, 41.5% (628 of 1513) occurred in prior CABG group and 33.0% (1221 of 3695) in non‐CABG treated group. The results were OR = 1.45% and 95% CI: 1.28–1.64 (*p *< .00001), with no heterogeneity (*I* ^2^ = 0.0%, *p* = .47; Figure 2C).

#### 3.2.5 Moderate or severe calcifications

A total of six trials reported the degree of calcification in both groups, which involved 10 905 patients. Figure 2 includes data on the moderate or severe calcifications in both groups. Pooled data analysis revealed 4613 calcification (overall rate 42.3%). Regarding treatment, calcification occurred in 60.0% (1447 of 2412) of prior CABG‐treated patients and 37.3% (3166 of 8493) of non‐CABG‐treated patients. The overall OR was 2.91 (95% CI: 2.19–3.86, *p* < .00001), and a high degree of heterogeneity was found among these studies (*I* ^2^ = 83%, *p* < .0001; Figure 2D).

#### 3.2.6 Bending

Only three studies involving a total of 1211 patients reported 4613 bending (overall rate 42.3%) in both groups. Regarding treatment, bending occurred in 53.6% (425 of 793) of prior CABG‐treated patients and 26.3% (786 of 2987) of non‐CABG‐treated patients. The pooled outcomes revealed the OR was 2.91 (95% CI: 2.19–3.86, *p* < .00001), and no heterogeneity was found for bending incidence(*I* ^2^ = 0.0%, *p* = .8; Figure 2E).

#### 3.2.7 Lesion length

Lesion length >20 mm has been well recognized as an unfavorable characteristic in angiographic. Four studies reported this characteristic, which involved 5782 patients and 3553 events in both groups (overall rate 61.4%). Among these patients, 67.8% (736 of 1085) occurred in the prior CABG treated group and 60.0% (2817 of 4697) in the non‐CABG treated group. The pooled outcomes revealed the OR was 1.59 (95% CI: 1.10–2.29, *p* = .01), with high heterogeneity (*I* ^2^ = 83%, *p* = .0004; Figure 2F).

#### 3.2.8 In‐stent CTO

In‐stent CTO was reported in five studies from 8903 patients and 1242 (overall rate 14.0%) was found. However, no significant difference was found between the two groups: per treatment, the event rate was 14.3% (304 of 2120) in the prior CABG group and 13.8% (938 of 6783) in the non‐CABG group. The pooled OR value was 0.99 (95% CI: 0.86–1.14, *p* = .88; Figure 2G), and there was no heterogeneity (*I* ^2^ = 0.0%, *p* = .44).

#### 3.2.9 Lack of interventional collaterals

The outcome occurred in 2013 events among 5208 participants (38.7%) from three studies, finding a negative result. Among these patients, 36.8% (557 of 1513) in the prior CABG treated patients and 39.4% (1456 of 3695) in non‐CABG treated patients (OR: 0.80, 95% CI: 0.55–1.15, *p* = .23), with high heterogeneity (*I* ^2^ = 78%, *p* = .01; Figure 2H).

#### 3.2.10 Inadequate distal landing zone

A total of 7266 patients were included in four studies reporting this event. And the incidence was 33.7% (2448 of 7266) in the overall rate. Compared with the treatment method, 44.1% (844 of 1914) in the prior CABG group and 30.0% (1604 of 5352) in the non‐CABG group. The pooled OR value was 1.95 (95% CI: 1.75–2.18, *p* < .00001; Figure 2I), and there was no heterogeneity (*I* ^2^ = 0.0%, *p* = .54).

#### 3.2.11 Distal cap at bifurcation

For bifurcation at the distal cap during undergoing coronary angiography, only three studies were included, reporting 1646 events among 5208 individuals (overall rate 31.6%), but finding a positive result. The incidence was significantly higher in patients with prior CABG (40.0%, 605 of 1513) when compared with non‐CABG patients (28.2%, 1041 of 3695). The pooled outcomes revealed the OR was 1.65 (95% CI: 1.46–1.88, *p* < .00001), with no heterogeneity (*I* ^2^ = 0.0%, *p* = .49; Figure 2J).

#### 3.2.12 Previously failed attempt

Previously attempted but failed were more common among harder CTO cases. Five studies provided this data, reporting 1677 events among 8573 individuals (19.6%). Regarding treatment, 18.0% (417 of 2313) occurred in the prior CABG group and 20.1% (1260 of 6260) in the non‐CABG treated group. The pooled outcomes revealed that the rate of previously failed attempt did not vary significantly between two groups (OR: 0.73; 95% CI: 0.48–1.11, *p* = .14; *I* ^2^ = 89%, heterogeneity *p* < .00001; Figure 2K).

#### 3.2.13 J‐CTO score

Four studies provided data regarding the comparison of J‐CTO score between the two groups which involved 7255 participants: 26.3% (1914 of 7255) in the prior CABG group and 73.7% (5352 of 7255) in the CABG‐naïve group. The score was significantly higher in the prior CABG group when compared with the non‐CABG group (2.74 ± 1.24 vs. 2.02 ± 1.26; *p* < .001). The pooled SMD value was 0.52 (95% CI: 0.42–0.63, *p* < .00001; Figure 2L), with moderate heterogeneity (*I* ^2^ = 65%, *p* = .04).

## 4 COMPLICATION

### 4.1 Perforation with need for intervention

**Figure 3:**
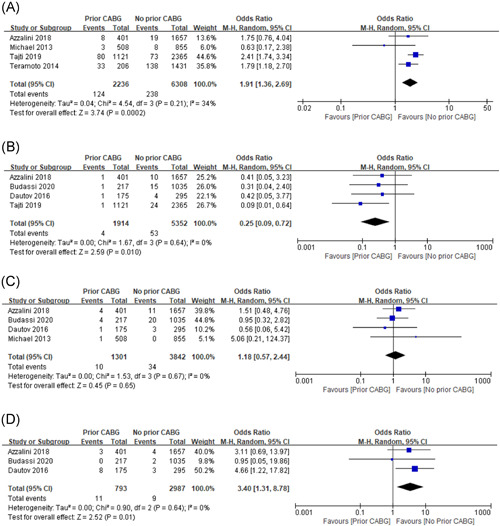
Forest plot for: (A) perforation with need for intervention; (B) tamponade; (C) major bleeding; (D) contrast‐induced nephropathy

8544 patients from four studies were included in the analysis of this event and the incidence was 4.24% (362 of 8544) in the overall rate. For the treatment method, the perforation incidence was significantly higher in the prior CABG group (5.55%, 124 of 2236), as compared with the non‐CABG group (3.77%, 238 of 6308). The pooled OR value was 1.91 (95% CI: 1.36–2.69, *p* = .0002), with lower heterogeneity (*I* ^2^ = 34%, *p* = .21; Figure 3A).

### 4.2 Tamponade

A total of four studies reported the incidence of tamponade, which involved 7266 participants and 57 events (0.78%). The pooled results indicated that the non‐CABG group may have a higher incidence of tamponade (0.99%, 53 of 5352), when compared with the prior CABG group (0.21%, 4 of 1914), during the perioperative period of CTO PCI (OR: 0.25, 95% CI: 0.09–0.72, *p* = .01) with no heterogeneity across studies (*I* ^2^ = 0.0%, *p* = .64; Figure 3B).

### 4.3 Major bleeding

A total of four studies were included for the major bleeding, which involved 5143 patients and 44 events (0.86%). And no heterogeneity was found for the incidence (*I* ^2^ = 0.0%, *p* = .67). Furthermore, there was no significant difference between the prior CABG (0.77%, 10 of 1301) and non‐CABG group (0.89%, 34 of 3842) regarding major bleeding (OR: 1.18, 95% CI: 0.57–2.44, *p* = .65; Figure 3C).

### 4.4 Contrast‐induced nephropathy

Only 20 events among 3780 patients from three studies analysis this event and get a positive result. The prior CABG treatment (1.4%, 11 of 793) was significantly associated with higher risks of contrast‐induced nephropathy, compared to non‐CABG treatment (0.30%, 9 of 2987). And the pooled OR value was 3.40 (95% CI: 1.31–8.78, *p* = .01), with no heterogeneity (*I* ^2^ = 0.0%, *p* = .64; Figure 3D).

## 5 DISCUSSION

Over the past years, CTO‐PCI has received much attention as one of the major frontiers of interventional cardiology, and advisory documents underlining technical and organizational aspects have been published.

Management of CTO by PCI or CABG or medical therapy has been controversial for a very long time.^16^ The summary statistic shows that of these patients, 11% undergo PCI, 40% undergo CABG, and 49% had medication. In patients without CTO, 36% underwent PCI, 28% underwent CABG, and 35% had medication.^17^

Some experts prefer that CABG should be prioritized to patients with CTO because patients with CTO often have high SYNTAX scoring and also have a high probability of combining multiple comorbidities.^18^ Multiple risk factors make patients highly variable in clinical and lesions characteristics. CABG has the capability to offer complete revascularization for both proximal and distal vessels, dating from two trials by DECISION‐CTO and EuroCTO.^19^, ^20^ In addition, as technology advances in recent years, the long‐term patency rate of graft vascular are higher. Not only the left internal mammary artery instead of LAD is very efficient, but also the saphenous vein long‐term patency of CTO is developed with an average of 75%, which brings a better outcome for CABG long‐term follow‐up.^17^

However, the use of PCI or CABG as the first choice to treat CTO is still a topic of debate.^21^ PCI of native‐vessel CTO for patients with prior CABG is often the preferred revascularization strategy compared with redo CABG. Repeat CABG has often been related to a greater risk of adverse events than initial CABG. However, CTO‐PCI for previous CABG patients is also difficult for interventional cardiologists that many scores and studies regard previous CABG as an independent risk factor for CTO‐PCI.

In our meta‐analysis, 13 439 CTO patients from eight studies were included, which contained 3349 (24.9%) pCABG patients and 10 090 (75.1%) nCABG patients. The main findings of our meta‐analysis demonstrated that: (1) patients with a history of CABG consist of a significant fraction in those undergoing CTO‐PCI that one in every four patients had prior CABG; (2) compared to nCABG patients, pCABG patients were older and had more multiple comorbidities; (3) patients with previous CABG had higher angiographic complexity; (4) the two groups complication rates were comparable.

First, we analyzed the characteristics of patients‐ rather than lesion‐specific factors between patients with or without CABG. Compared to non‐CABG patients, prior CABG patients were older, more frequently male, and had more comorbidities such as diabetes mellitus, hypertension, and dyslipidemia. Also, they had a more previous myocardial infarction and PCI history. However, a pleasant surprise is that they had lower current smokers, which may attribute to better patient education and self‐management.

Next, we analyzed the CTO target vessel location in both groups. Some multicenter CTO registry studies have reported that in the whole CTO, 47% CTO was located in the RCA, 20% in the LAD, 16% in the LCX or 17% multiple locations. Consistent with previous trials, our meta‐analysis showed that the RCA was the most common CTO vessel no matter the CABG history. Although the LCX was more frequent CTO target vessel in post‐CABG patients, whereas the LAD was more frequently located in CABG‐naïve patients, presumably because of the high patency rates of the left internal mammary artery. Furthermore, in the past studies, it has been questioned whether the CTO target vessel location is associated with the success rate of recanalization. And some previous studies have proposed that LCX‐CTO is related to a lower recanalization success rate, less efficiency, and higher complications rate.^22^ Even the PROGRESS‐CTO (Prospective Global Registry for the Study of CTO Intervention) score has included LCX‐CTO as an independent predictor of technical failure.^23^ Although, a more recent multicenter study reported similar success rates in all arteries, the patients with successful recanalization of LCX‐CTO showed a higher cardiac long‐term mortality compared to other arteries.^24^ The lower success rate for LCX‐CTO is likely associated with the frequent tortuosity and less interventional collaterals of this vessel.

Knowledge of potential caveats in treating CTO might cause patient selection; however, the decision of revascularization is done based on clinical need, not anatomical characteristics. Therefore, we further analyzed the lesion morphology of CTO target vessel.

The CTO lesion begins with the proximal cap. The proximal cap morphology, clarity, and whether there is a side branch at proximal entry decided the wire to probe the occlusion.^25^ The CTO stump has two types which are tapered tip stump and blunt stump. While there are two types of pathological vascular channels that extend the CTO occluded segment which are endothelialized microchannel and microcapillary. The endothelialized microchannel, termed histologically recanalized segment, is a 160–230 µm diameter neovascularization that connects the occluded segment from the proximal to distal cap. The microcapillary, termed nonrecanalized segment, is a <100 µm diameter capillary that passes into the vasa vasorum or small side branch which cannot span the occluded segment from the proximal to distal cap. The tapered tip stump CTO is more likely associated with a histologically recanalized segment and less likely to have a major side branch. In contrast, the blunt stump CTO is more likely to have a non‐recanalized microcapillary, which means it will be more difficult to open the CTO lesion.^26^ In our meta‐analysis, patients with previous CABG undergoing CTO‐PCI have a more blunt stump and proximal cap ambiguity, but the side branch at proximal entry has no significant difference between the two groups. The association between CABG and accelerated atherosclerosis progression was clearly elucidated in many studies. Especially in the proximal vessel, the competitive flow generated by the graft vascular may play a dominant role. Moreover, it has been hypothesized that the exposure of the distal cap to arterial pressure from the graft may even promote adverse remodeling and blunt rather than tapered morphology.

Once the wire crossed the proximal cap, it will enter the body of the CTO occluded segment. In each body of the CTO lesion, its difficulty and complexity depending on the degree of calcification, tortuosity, and length. In particular, the length of the occluded segment is the most significant factor to decide the success of crossing a CTO. The longitudinal continuity of microchannels spans around 85% of the total CTO length.^27^ These histological characteristics provide the basis for multiple CTO‐PCI algorithms. Some experienced operators believe that length greater than 20 mm was a major predictor of procedural failure to cross the occlusion segment than calcification, tortuosity, and blunt stump. In our meta‐analysis, prior CABG is associated with more severe calcification, more tortuosity, and longer length of CTO, due either to the shrinkage or distortion of the occluded bypass graft vessel after CABG. Blood stasis and low shear stress resulting from the competitive flow between native arteries and bypass graft may also consist of the mechanism for greater calcification in the native vessel. These characteristics are all considered as challenges in the CTO‐PCI process.

Collateral channels originate as arterioles connecting the vascular beds of visible coronary arteries. With the developed chronicity of a CTO, these small arteriole collaterals undergo remodeling to become muscular arteries known as arteriogenesis.^28^ For appropriate interventional collaterals, the collateral channels can be septal arteries, epicardial connections but also can be graft vascular (either arterial or venous).^29^ By using the backdoor to pass the guidewire from the donor artery via a collateral channel to penetrate the distal cap, the success rate of CTO‐PCI has increased to >90% in the hands of CTO masters with a proper retrograde approach.^30^ In our meta‐analysis, the number of interventional collaterals has no significant difference between the two groups. Whereas on the other hand, bypass grafts can be used as collateral channels (even when occluded) to facilitate interventional devices via the retrograde approach. Data showed the most commonly used collaterals for the retrograde approach in prior CABG patients were septal collaterals (43%), followed by epicardial collaterals (34%), saphenous vein graft (30%), and left internal mammary artery grafts (3%).^13^

Inadequate distal landing zone was defined as a distal vessel segment with a diameter less than 2.0 mm or with a diffuse lesion.^13^ An inadequate landing zone represents an unfavorable feature for antegrade true‐to‐true lumen approach and dissection/re‐entry techniques. However, bifurcation at the distal cap represents a favorable feature for retrograde approach. In our meta‐analysis, for inadequate distal landing zone, prior CABG is related to significantly higher incidences, and for a distal cap at bifurcation, to our pleasant surprise, the incidence was significantly higher in patients with prior CABG which means they will have a good chance for retrograde approach.^31^

These characteristics of CTO target vessel are also taken into account by risk scores for predicting technical success rate, such as the J‐CTO (multicenter CTO registry in Japan) score.^32^ Currently, the J‐CTO score is the most broadly used score to predict successful guidewire crossing through native CTO occluded segment within 30 minutes. The J‐CTO score included five characteristics of the CTO, which are related to procedural challenges: blunt stump, moderate/severe calcifications, >45° bending, length >20 mm, and retry CTO‐PCI. And the score defined all lesions into four difficulty groups: easy (J‐CTO score = 0), intermediate (score of = 1), difficult (score of = 2), and very difficult (score of ≥ 3). In our meta‐analysis, the score was significantly higher in the prior CABG group (2.74 ± 1.24), when compared with the non‐CABG group (2.02 ± 1.26) (*p* < .001). The scoring model not only objectively evaluates the clinical and anatomic complexity but also quantitatively measures the difficulty and probability of CTO‐PCI revascularization success rate, which can guide the interventionalist to make proper clinical decisions for each CTO patient.

The CTO‐PCI is considered as one of the last frontiers for interventional cardiologists. Although the success rate for it has improved year by year with the development of new techniques and available devices, the procedural complication is still an unavoidable risk.^33^ In our meta‐analysis, we analyzed four main complications those are perforation with the need for intervention, tamponade, major bleeding, and contrast‐induced nephropathy. And to our pleasant surprise, the procedural complications are rare in both groups and the rates of complications in the prior CABG group remain comparable to patients in the non‐CABG group. Notably, perforation with the need for intervention, even tamponade, is a fatal complication for CTO‐PCI.^34^ Our meta‐analysis demonstrates that CTO‐PCI in prior CABG patients is related to a higher rate of perforation, but a lower rate of pericardial tamponade. A possible explanation of this result may be attributed to the commonly held belief that the potential protective effect of pericardial adhesion in prior CABG patients.^35^ However, CABG is not immune for patients to tamponade, as previously thought. First, the pericardium may restore by itself after cardiac surgery, especially in young patients.^36^ Furthermore, the pericardium is remained an open state creating a pseudo‐pericardial space commonly.^37^ In this space, the risk of pericardial tamponade is still present, which occurs in approximately 40% of all CABG patients with coronary artery perforation.^38^ Last but not least, it is well known that a dry tamponade may happen with localized fluid collection around one cavity in a patient with prior CABG. The loculated hematomas can compress the atria or ventricles, potentially progressing to cardiogenic shock. Even more dire is the dry tamponade without detection of relevant pericardial effusion at common echocardiography. Considering the potential catastrophe of coronary perforations for patients with CABG history, the experts recommended immediate drainage or surgery treatment.

## 6 LIMITATIONS

Our study has some limitations. First, all the studies entered our review were observational study, because no randomized data are present. With all the inherent bias ascribed to this kind of design, the results might be affected by residual and unmeasured confounders. Second, the meta‐analysis pooled the data at the study level and not patient level, which prevented comprehensive assessment to identify patient characteristics. For lacking patient‐level data, the clinical characteristics and angiographic are precluded statistical adjustment, which may be associated with CTO difficulty in PCI and procedural complication. Third, to make a comparison of the CTO‐PCI complications, the success rates of prior CABG and no CABG group should be at the same level or a comparable level. A readily given‐up procedure means less risk and no complication. On the other hand, the operators might be biased to adopt the aggressive techniques for patients who have undergone CABG, with a false security that the risk of catastrophic complications (such as tamponade) is lower. These issues might confound the relationship between success rates and procedural complications. The method of meta‐regression may solve this question. However, the meta‐regression requires the number of studies included in meta‐analysis not too small, and more than 10 are recommended. Otherwise, the selected influencing factors are extremely unstable. Finally, publication bias is possible that more experienced centers and higher volume might be more likely to publish their outcomes.

## 7 CONCLUSIONS

In the previous study, successful PCI for CTO was related to increasing living quality in both patients with and without CABG history. Furtherly, interaction analyses indicated a similar revascularization profit in both groups. These results extend our thinking about benefits related to successful CTO revascularization for the high‐risk patient subgroup with prior CABG and highlight the value to provide PCI for them. According to our results, CTO‐PCI is frequently performed in patients with prior CABG. Nevertheless, CTO‐PCI for patients who have undergone CABG is still a complex intervention and is related to higher angiographic challenges such as blunt stump, proximal cap ambiguity, severe calcifications, bending and lesion length >20 mm. Even in the PROGRESS scores, the CABG history represents an independent risk of difficulty. Taking those specificities into account, some experts encourage that the retrograde approach should be accepted more widely, and performed appropriately.^31^ And to our pleasant surprise, the rate of complications in the prior CABG group remains comparable to the non‐CABG group, which makes interventionalists motivated to update their techniques and equipment.

## CONFLICT OF INTERESTS

The authors declare that there are no conflict of interests.

## AUTHOR CONTRIBUTIONS

Yuchen Shi, Songyuan He, and Jinghua Liu conceived the study and designed the protocol. Yuchen Shi and Songyuan He integrated the data and drafted the manuscript. Wen Jiana and Xueqian Shen were responsible for the study selection, data extraction, and assessment of study quality. Jesse Luo and Jinghua Liu revised the manuscript critically. All authors read and approved the final manuscript.

## Data Availability

Data available on request from the authors. The data that support the findings of this study are available from the corresponding author upon reasonable request.
